# Solid-state ensemble of highly entangled photon sources at rubidium atomic transitions

**DOI:** 10.1038/ncomms15501

**Published:** 2017-05-26

**Authors:** Robert Keil, Michael Zopf, Yan Chen, Bianca Höfer, Jiaxiang Zhang, Fei Ding, Oliver G. Schmidt

**Affiliations:** 1 Institute for Integrative Nanosciences, IFW Dresden, Helmholtzstraße 20, 01069 Dresden, Germany; 2 Institut für Festkörperphysik, Leibniz Universität Hannover, Appelstraße 2, 30167 Hannover, Germany; 3 Merge Technologies for Multifunctional Lightweight Structures, Technische Universität Chemnitz, 09107 Chemnitz, Germany

## Abstract

Semiconductor InAs/GaAs quantum dots grown by the Stranski–Krastanov method are among the leading candidates for the deterministic generation of polarization-entangled photon pairs. Despite remarkable progress in the past 20 years, many challenges still remain for this material, such as the extremely low yield, the low degree of entanglement and the large wavelength distribution. Here, we show that with an emerging family of GaAs/AlGaAs quantum dots grown by droplet etching and nanohole infilling, it is possible to obtain a large ensemble of polarization-entangled photon emitters on a wafer without any post-growth tuning. Under pulsed resonant two-photon excitation, all measured quantum dots emit single pairs of entangled photons with ultra-high purity, high degree of entanglement and ultra-narrow wavelength distribution at rubidium transitions. Therefore, this material system is an attractive candidate for the realization of a solid-state quantum repeater—among many other key enabling quantum photonic elements.

## Abstract (web summary)

Scalable and integratable sources of entangled-photon pairs are an important building block for quantum photonic applications. Here, Keil *et al*. demonstrate that an ensemble of droplet-etched gallium arsenide quantum dots can emit polarization-entangled photons with almost 100% yield.

Solid-state sources that emit single pairs of entangled photons are a key element in quantum information technology. Polarization-entangled photons from atomic cascades were first used to test Bell's inequality^1^,^2^, but demonstrating scalable applications with single atoms is clearly a technological challenge. In 1988, Shih and Alley^3^ reported that the photon pairs generated from spontaneous parametric down conversion^4^ are polarization entangled and can violate Bell's inequality, opening the door for various polarization-entanglement-based experiments. However, spontaneous parametric down conversion sources are characterized by Poissonian statistics, that is, a tradeoff has to be made between the source brightness and the multiphoton emission probability. This lack of on-demand single-photon generation fundamentally limits their applications in complex quantum protocols.

Semiconductor InAs/GaAs quantum dots (QDs) grown by the Stranski–Krastanov method are among the leading candidates for the deterministic generation of polarization-entangled photons^5^,^6^,^7^,^8^,^9^,^10^. As proposed by Benson *et al*.^5^, the cascaded emission in single QDs from the biexciton 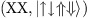 to the ground state via the intermediate exciton states 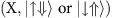 produces polarization-entangled photon pairs 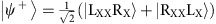, where R and L denote right- and left-handed circular polarization, respectively. In real InAs/GaAs semiconductor QDs, however, the anisotropy in strain, composition and shape reduces the QD symmetry and mixes the two bright exciton states, resulting in two non-degenerate bright exciton states 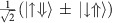 split by the fine structure splitting (FSS)^11^. The final two-photon state has a time-varying form 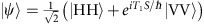, where *T*_1_ is the radiative lifetime of the exciton, *S* the FSS and H and V horizontal and vertical linear polarization, respectively^12^. To reduce the phase shift between the |HH〉 and |VV〉 two-photon components and to obtain a high degree of entanglement, the experimental strategies are to reduce the FSS *S* and/or the exciton lifetime *T*_1_.

This is unfortunately no easy task. In the last decade there have been extensive efforts to generate entangled photons with InAs/GaAs QDs. The probability of finding suitable QDs in an as-grown sample is <10^−2^ (refs 13, 14), thus necessitating the use of post-growth tuning techniques (such as thermal annealing, the optical Stark effect, magnetic, electric and strain fields) to eliminate the FSS^15^. On the one hand, the fact that every single QD needs to be independently engineered imposes a great challenge for the practical application of QD-based devices. On the other hand, due to the electron–nuclear spin hyperfine interactions^16^,^17^, the degree of entanglement of InAs/GaAs QD-based sources is generally low even at zero FSS. The best result so far yields an entanglement fidelity *F*=0.82 and concurrence *C*=0.75 (ref. 18). Alternatively, one can reduce the exciton lifetime *T*_1_ by using the Purcell enhancement in a cavity, or perform time gating before a significant phase shift *T*_1_*S*/*ħ* between |HH〉 and |VV〉 takes place. The practical implementation of the former requires a simultaneous Purcell enhancement of both X and XX emissions^6^, which is a nontrivial task. The latter discards a large portion of photons and reduces the source brightness significantly. Applying post selection, fidelities up to *F*=0.86 have been achieved using an InAsP QD containing InP nanowire^19^.

Based on the above discussion, it is possible to obtain a large ensemble of QD-based polarization-entangled photon emitters by simultaneously incorporating a highly symmetric confinement potential, a short radiative lifetime and a weak electron–nuclear spin hyperfine interaction. The first attempt was reported by Juska *et al*.^13^, where arrays of symmetric In_0.25_Ga_0.75_As_1−*δ*_N_*δ*_ QDs were grown on the GaAs (111)B surface. They were able to obtain areas with an impressive 15% of entangled photon emitters with fidelities *F* in the range of 0.5 up to 0.72. Although the FSS is consistently below 4 μeV for these novel QDs, the exciton lifetime is quite long (1.8±0.6 ns). Nevertheless, the violation of Bell's inequality was recently shown in electrically injected pyramidal QDs^20^. Kuroda *et al*.^21^ demonstrated the generation of entangled photons (with fidelity up to *F*=0.86) using highly symmetric GaAs/AlGaAs QDs grown on the GaAs (111)A surface by droplet epitaxy. Although the exciton lifetime is short (560 ps), the FSS are relatively large (with a mean value of 10±5 μeV) and the hyperfine interaction of the exciton with nuclear spins is significant in this system^21^.

In this work, we show that a large ensemble of as-grown polarization-entangled photon emitters can be obtained, using an emerging family of GaAs/AlGaAs QDs grown by droplet etching and nanohole infilling. These QDs exhibit very small FSS (with a mean value of 4.8±2.4 μeV) and short radiative lifetime (*T*_1_<220 ps). Under pulsed resonant two-photon excitation, a coherent excitation of the biexciton state can be achieved (with pronounced Rabi oscillations up to 7*π*), and all measured QDs emit single pairs of entangled photons with ultra-high purity and high degree of entanglement (fidelity *F* up to 0.91, concurrence *C*=0.90). The QDs presented in this work offer a deterministic wavelength control and ultra-narrow wavelength distribution, specifically tailored to match the optical transitions of rubidium. Thereby, we envision a hybrid quantum repeater that incorporates QD-generated entangled photon qubits interfaced with a rubidium vapour-based quantum memory.

## Results

### Sample growth

**Figure 1:**
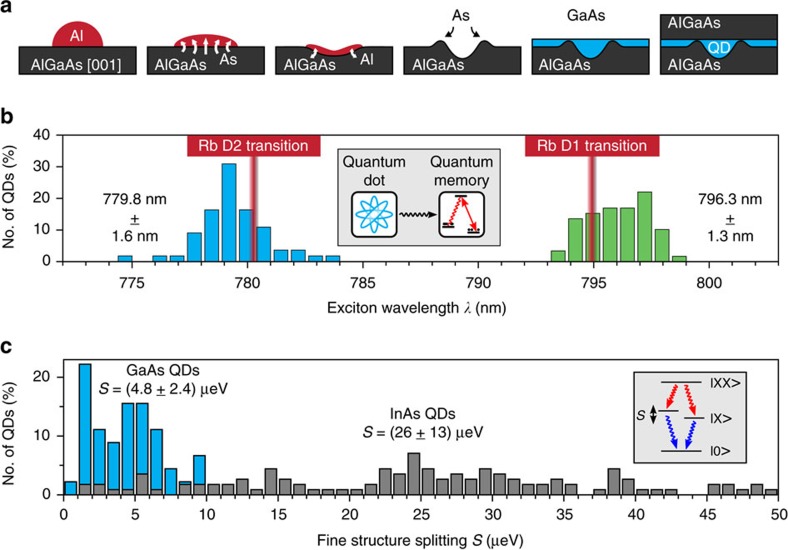
Growth and properties of highly homogeneous GaAs/AlGaAs quantum dots. (**a**) Processing steps during the growth of GaAs/AlGaAs quantum dots (QDs): Al is deposited under low arsenic pressure, forming droplets on the surface of AlGaAs grown on a GaAs (001) substrate. The concurring dissolution of As through the droplets and diffusion of Al towards the substrate (illustrated by arrows) induces the formation of nanoholes with high symmetry. In a following annealing step the structures crystallize under a re-established As atmosphere. Then, the nanoholes are filled with GaAs and subsequently overgrown by AlGaAs to obtain isolated QDs with three-dimensional carrier confinement. (**b**) Exciton emission wavelength distribution for two different samples with GaAs infilling amounts of 2 nm (blue) and 2.75 nm (green) for more than 50 dots measured on each sample. Red markers indicate the rubidium D1 and D2 transition lines at 794.9 and 780.2 nm, respectively. Inset: sketch of envisioned interface between entangled photons from a QD and an atomic quantum memory based on the Raman scheme^53^. (**c**) Occurrence of the exciton fine structure splitting, comparing the GaAs/AlGaAs QDs (blue) with InAs/GaAs QDs (grey). Inset: scheme of the biexciton (XX) decay indicating the spin-related fine structure splitting *S* between the intermediate exciton states (X).

The QDs presented in this work are fabricated by solid-source molecular beam epitaxy. The *in situ* droplet etching^22^,^23^ is used to create self-assembled nanoholes with ultra-high in-plane symmetry^24^,^25^, which are subsequently filled and capped to obtain embedded solid-state quantum emitters^26^,^27^. Figure 1a shows a sketch of the processes involved in the QD formation. The initial point is a GaAs (001) substrate that has been deoxidized and overgrown with a GaAs buffer layer followed by 200 nm of Al_*x*_Ga_1−*x*_As. During the growth As_2_ is provided by a cracker cell running at 650 °C. First, Al is deposited under low arsenic pressure (<10^−8^ mbar), forming droplets on the surface at 630 °C. Driven by concentration gradients, the concurring dissolution of As through the droplets and diffusion of Al towards the substrate induces the formation of nanoholes with high symmetry. In a following annealing step at 630 °C the structures crystallize under a re-established As atmosphere of 10^−7^ mbar. Then, the nanoholes are filled with GaAs and subsequently overgrown by Al_*x*_Ga_1−*x*_As to obtain the isolated QDs with three-dimensional carrier confinement.

Envisioning a hybrid QD–atomic interface as a promising solid-state quantum memory^28^,^29^, it is desirable to match the QD emission with atomic transitions, illustrated by the inset in Fig. 1b. For this purpose several samples with varying GaAs infilling amounts have been grown, targeting the Rb D1 and D2 transition lines at a wavelength of 794.9 and 780.2 nm, respectively. Figure 1b shows the exciton wavelength distribution for two different samples with 2 nm (blue) and 2.75 nm (green) GaAs deposited at a growth rate of 0.47 and 0.5 μm h^−1^, accordingly. The statistics on more than 50 QDs across an area of 1 cm^2^ on each sample show an unprecedented control on the central emission wavelengths, with mean values of 779.8±1.6 nm and 796.3±1.3 nm. The wavelength distributions, or the so-called inhomogeneous broadenings, are among the narrowest for semiconductor QDs and are about 5 times smaller than that of a typical self-assembled InAs/GaAs QD sample. A similarly narrow inhomogeneous broadening can be observed in pyramidal QDs^20^,^30^.

Together with the high homogeneity, the QDs also exhibit high symmetry due to the negligible intermixing and a virtually strain-free interface between GaAs and AlGaAs. Previous work suggests that a reduction of the amount of deposited Al and an increase of the deposition rate can enhance the nanohole symmetry^25^. Following this trend, a single pulse of 0.09 nm excess Al at a growth rate of 0.8 μm h^−1^ (corresponding to AlAs growth) was used for our samples. The optimized growth protocols lead to high-quality QDs. Figure 1c shows the statistical distribution of the FSS for the GaAs/AlGaAs QD sample studied in this work (blue) and for a typical InAs/GaAs QD sample grown by partial capping and annealing (grey). The total number of measured dots is 45 and 114, respectively. The GaAs QDs feature an average FSS of only 4.8±2.4 μeV that is among the best values reported so far^13^,^21^,^25^. With these superior spectral properties, the investigated samples are promising candidates for the generation of polarization-entangled photons.

### Resonant excitation of the biexciton

**Figure 2:**
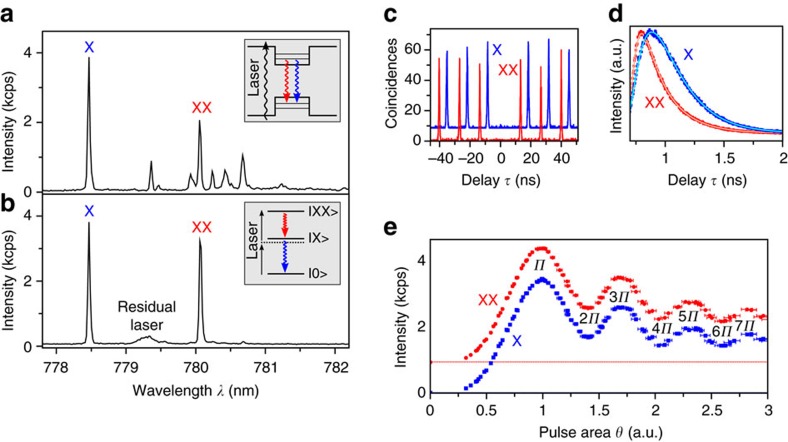
Resonant excitation of the biexciton state in GaAs/AlGaAs quantum dots. (**a**) Quantum dot (QD) emission spectrum for pulsed above-band excitation. The dominant exciton (X) and the biexciton (XX) transition are observed, which are spectrally close to other excitonic species. (**b**) Resonant excitation of the XX state using a two-photon excitation scheme. This efficient and coherent pumping mechanism results in a pure spectrum featuring primarily the XX–X cascade. The centric residual laser signal is strongly suppressed by using notch filters. (**c**) Intensity-autocorrelation measurement of the XX and X transition showing the coincidences plotted over the delay time *τ*. Very pure single-photon emission is confirmed by 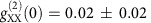 and 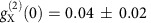. (**d**) Measurement of the fluorescence lifetime *T*_1_ for the XX and X state. The respective fit functions (solid lines) denote the convolution between an exponential decay and the detector response function. Short radiative lifetimes of *T*_1,XX_=112 ps and *T*_1,X_=134 ps are determined. (**e**) Fluorescence intensities of the XX and X emissions as a function of the pulse area θ, obtained by excitation laser power-dependent measurements. Rabi oscillations up to 7*π* are observed for both transitions. An offset is added to the XX values for better visibility, with the dotted lines indicating zero intensity.

The major challenge to the realization of entangled photon pair emission from droplet-etched GaAs/AlGaAs QDs is the effective excitation of the biexciton (XX) state. In pyramidal GaAs/AlGaAs QDs the biexciton has been observed^31^, as well as in QDs based on GaAs/AlGaAs quantum well thickness flucutations^32^,^33^. So far there are only few reports about the observation of a biexciton in GaAs/AlGaAs QDs grown by local droplet etching^21^,^27^, presumably due to the low internal population probability under nonresonant excitation. Due to the optimized growth process we are able to observe strong XX emissions even with above-band excitations. We select a QD from the sample emitting close to the Rb D2 transition (∼780.2 nm) and excite it by pumping the surrounding higher-bandgap AlGaAs with a pulsed laser. The resulting spectrum (Fig. 2a), which is relatively clean in a broad range, shows several different excitonic transitions: The transition with the highest intensity is the exciton emission (X) at *λ*=778.5 nm. Among several red-shifted transitions, the XX emission is the strongest (*λ*=780.1 nm).

To efficiently excite and to coherently drive the XX transition, we pump the two-photon resonance of the XX state by using a pulsed laser that lies spectrally in between the X and XX transitions. This excitation scheme has already been proven very effective in case of InAs/GaAs QDs^34^. Making use of tunable notch filters we can effectively suppress the laser background. Hence, a very pure spectrum showing mostly the XX and X emissions can be observed (Fig. 2b). The integrated intensities are the same for both emissions, strongly indicating a close to unity efficiency for the cascaded emission process^34^.

To obtain the evidence of pure single-photon emissions from both XX and X, we perform an autocorrelation measurement at *π*-pulse excitation using a standard Hanbury Brown and Twiss setup and the results are shown in Fig. 2c. The autocorrelation function *g*^(2)^(*τ*) plotted over the photon arrival delay *τ* shows a clear absence of counts at zero delay and proves the ultra-high purity single-photon emission. The background-corrected correlation function is measured to be 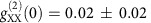 for XX and 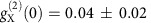 for X.

Next, we measure the luminescence lifetime *T*_1_ by recording an intensity correlation between the excitation laser pulse and the arrival time of the photons (see Fig. 2d). The XX shows a simple exponential decay, which is fitted taking into account the convolution with the detector response function. The X decay shows a longer rise time since the state has to be populated first by the decay of the XX state. The extracted lifetimes are *T*_1,XX_=112 ps and *T*_1,X_=134 ps, and these are among the lowest values recorded for as-grown semiconductor QDs. The ideal lifetime-limited linewidth of the exciton emission is therefore Δ*E*=4.9 μeV, close to the mean value of the FSS in our sample.

To further evaluate the resonant two-photon excitation scheme, we record the intensity of XX and X photons while changing the excitation power. The result is summarized in Fig. 2e by plotting the intensity over the pulse area θ that is proportional to the square root of the excitation power. Clear Rabi oscillations are observed, which are oscillations of the intensity due to a coherent rotation on the Bloch sphere between the ground state |0〉 and the excited state |XX〉. The abscissa is normalized in units of *π* to the first maximum of the XX intensity, where the pulse area is equal to *π*. Intensity oscillations up to 7*π* are observed. The mean intensity is decreasing for higher excitation powers, which may be caused by several different factors like chirp in the excitation pulse or scattering processes in the QD^34^. Increasing the power also leads to an increase in the oscillation frequency. This is a fingerprint of the two-photon excitation process in clear contrast to one-photon resonant excitation, where the frequency remains constant.

### Evaluating the degree of entanglement

After realizing an efficient coherent control over the XX decay in GaAs QDs we now evaluate the degree of entanglement in the polarization of the emitted photons. A QD with a FSS of *S*=2.3 μeV is chosen in the experiment, since it represents a large portion (∼22%) of QDs in the sample (see Fig. 1c). The QD is excited with *π*-pulses for an efficient preparation of the biexciton state. To measure the degree of polarization correlation we send the stream of XX and X photons onto a 50:50 beam splitter. Each subsequent signal arm contains a quarter-wave plate, a half-wave plate and a polarizer to select the polarization in an arbitrary basis. After spectral selection of XX and X photons in the first and second signal arm, respectively, they are sent to single-photon detectors. Coincidence counting hardware is used to obtain the second-order correlation function 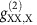 between XX and X photons for the selected polarization direction.

**Figure 3:**
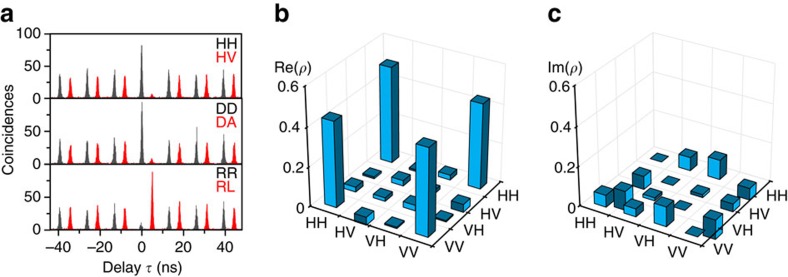
Degree of entanglement from a quantum dot with finite fine structure splitting. (**a**) Cross-correlation measurements between the biexciton and exciton emission for co- and cross-polarized photons in the rectilinear (H, horizontal; V, vertical), diagonal (D, diagonal; A, antidiagonal) and circular (R, right-handed; L, left-handed) polarization bases. For better visibility an offset in the delay time *τ* is added in the cross-polarized case. From these measurements on a quantum dot (QD) with a finite fine structure splitting of *S*=2.3 μeV a fidelity *F*=0.88±0.03 to the state 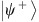 is deduced. (**b**,**c**) Real (**b**) and imaginary (**c**) part of the two-photon density matrix as reconstructed from 16 correlation measurements of the same QD by employing the maximum likelihood technique. The fidelity and concurrence extracted from this matrix are *F*=0.91 and *C*=0.90, respectively.

Figure 3a shows six cross-correlation measurements obtained for three bases of co-polarized and cross-polarized photons: the rectilinear (HV), diagonal (DA) and circular (RL) basis. As expected for an ideal entangled two-photon state 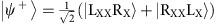, a strong bunching (antibunching) at *τ*=0 is observed for co-polarization (cross-polarization) in the rectilinear and orthogonal bases, whereas this behaviour is reversed for the circular basis set. The correlation contrast for a chosen basis set is given by^17^


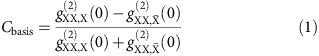


with 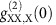 denoting the second-order correlation function at zero delay in collinear, and 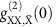 in orthogonal bases. For the three illustrated basis sets the following contrasts are obtained:


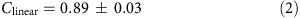



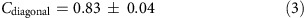



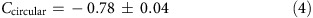


The fidelity *F* of the measured quantum state to the ideal state 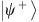 can then be obtained by^17^


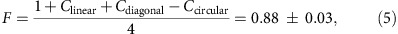


which exceeds the classical limit *F*=0.5 by more than 12 standard deviations.

A more comprehensive picture of the measured entangled two-photon state is given by the density matrix representation. We performed cross-correlation measurements for 16 different base combinations to account for the 16 unknown variables in the density matrix *ρ*. The measured values for *g*^(2)^(0) are then used to construct a density matrix following the procedure presented in ref. 35. Since the thereby obtained density matrix violates important basic properties like positive semidefiniteness, the maximum likelihood estimation is employed to find the appropriate density matrix that is the closest to the measured results. The resulting matrix is shown in Fig. 3, split into the real part (Fig. 3b) and imaginary part (Fig. 3c). The strongest features are observed in the outer-diagonal real-part matrix elements, which are close to 0.5, while all other elements are close to zero. This is in agreement with the expected entangled state 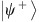 whose density matrix should have only non-zero values of 0.5 in the outer-diagonal elements. The small (but non-zero) real values in the off-diagonal elements indicate a weak spin scattering process in the QD. The finite imaginary off-diagonal values represent a small phase difference between |HH〉 and |VV〉, presumably caused by the joint effect of a finite FSS and an accumulated phase due to the optical setup. From this density matrix, we obtain a fidelity *F* (after background corrections^34^) to the state 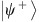 of


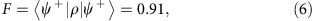


which is very close to the value of 0.88±0.03 obtained from the 6 cross-correlation measurements in Fig. 3a.

Another measure for nonclassical properties of a quantum state is the concurrence *C*^35^. Using the acquired density matrix, a value of *C*=0.90 (raw data without correction: *C*=0.81) is obtained. This is not only surpassing the best value measured for InAs/GaAs QDs with zero FSS^18^, but is also the highest value obtained for any QD entangled photon source so far. The high values for fidelity and concurrence are especially remarkable considering the finite fine structure splitting of *S*=2.3 μeV, which already significantly degrades the entanglement in case of InAs/GaAs QDs^17^,^36^.

**Figure 4:**
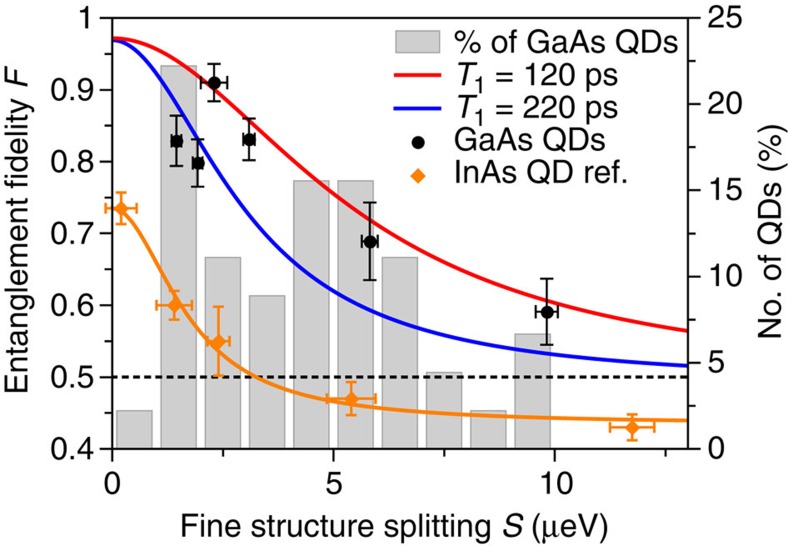
Entanglement fidelity of quantum dots with different fine structure splittings. The data points and traces illustrate the entanglement fidelity (left axis) and the grey histogram bars the occurrence of quantum dots (right axis), both plotted over the fine structure splitting *S*. All measured GaAs/AlGaAs quantum dots (QDs) from the sample (black circles) emit entangled photons with a fidelity above the classical limit of *F*=0.5 (dashed line) even for the largest values of *S*=9.8 μeV (*F*=0.59). The highest fidelity is measured to be *F*=0.91 at a nonvanishing *S*=2.3 μeV. For comparison, fidelity values of InAs/GaAs QDs taken from ref. 36 are plotted in orange, together with a Lorentzian fit. The fidelity uncertainties are obtained using error propagation and Poisson statistics for the coincidence counts. The uncertainties for *S* are determined by least square regression of polarization-dependent fluorescence measurements. Using a theoretical model, the fidelity *F*(*T*_1_, *S*) is plotted for two radiative lifetimes *T*_1_=120 ps (red) and *T*_1_=220 ps (blue), representing the range of all measured values for *T*_1_ in the sample. The applied model implies that both cross-dephasing and spin scattering processes are significantly suppressed in this material system. Together with the fine structure splitting distribution (grey histogram), the fidelity measurements strongly indicate that close to 100% of the QDs in the sample are polarization-entangled photon emitters.

Since the phase shift *T*_1_*S*/*ħ* between |HH〉 and |VV〉 states is significantly reduced due to the very short lifetime *T*_1_ in this system, we expect that the generation of entangled photons should be also possible for QDs with even higher values of *S*. Therefore, we select six dots representing the whole range of FSS measured in the sample. By measuring six cross-correlations in three basis sets for each dot, their entanglement fidelities *F* are obtained. Figure 4 shows the values of *F* plotted as a function of the FSS (black circles), overlaid on the FSS distribution in the sample (grey histogram). The data from Zhang *et al*.^36^ including a Lorentzian fit are shown as a reference for typical InAs/GaAs QDs (orange line). Remarkably, all of the measured dots show a clear signature of entangled photon emission with *F*>0.5. Even the QD with *S*=9.8 μeV, which represents the QDs with the largest FSS in our sample, shows a fidelity *F*=0.59±0.05. We want to highlight that the measured dots were not preselected according to certain conditions apart from their FSS. All measurements lead to the conclusion that nearly 100% of the QDs in this sample show entangled photon emission.

Another outstanding feature of this material system are the significantly higher fidelities compared with that of the typical InAs/GaAs QDs, mostly originating from the weak electron–nuclear spin hyperfine interactions in this type of QDs^16^,^17^,^37^. To better understand the obtained values, we plot two theoretical curves showing the fidelity over the FSS for radiative lifetimes of *T*_1_=120 ps (red curve) and *T*_1_=220 ps (blue curve), the typical range for the measured exciton lifetimes. We modelled the fidelity following the work by Hudson *et al*.^17^ that includes the influence of the FSS and lifetime *τ* as well as cross-dephasing and spin scattering:


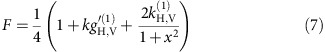


with


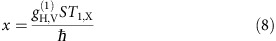


Here, *k* denotes the probability that the measured photon pairs originate from the dot. We estimate it to be *k*=0.97 due to the measured autocorrelation measurements presented in Fig. 2b. The factor 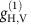 denotes the fraction of the QD emission that is unaffected by both cross-dephasing and spin-scattering processes, while 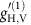 only considers spin-scattering processes. Since the presented data show no trend that would lead to *F*<0.5 for large FSS, we expect the influence of spin scattering processes on the entanglement to be negligible in this material system. Considering spin scattering due to the Overhauser field of the nuclear spins present in the dot, a spin-scattering time of *T*_SS_=15 ns can be assumed^38^. This is, however, two orders of magnitudes longer than the measured radiative lifetimes and therefore barely contributes to the degradation of the fidelity. On the other hand, in typical InAs/GaAs QDs this effect can be significantly stronger in case of high concentrations of spin-9/2 indium^38^, leading to much lower fidelity values for InAs/GaAs QDs in Fig. 4 (ref. 36). Since the fidelities at small FSS are very high for the GaAs/AlGaAs QDs, we neglect cross-dephasing processes in the model. It is clear that the trend in all our fidelity measurements can be well represented by the employed model.

## Discussion

In this work, we propose a new type of solid-state polarization-entangled photon source based on an emerging family of GaAs/AlGaAs QDs. These QDs can be grown with unprecedented wavelength control, ultra-small FSS and short radiative lifetime. The efficient and coherent excitation of the biexciton state in the GaAs/AlGaAs QDs is achieved by employing a resonant two-photon excitation scheme. The combination of a highly symmetric confinement potential, a short radiative lifetime and a weak electron–nuclear spin interaction in this material system enables entanglement fidelities up to *F*=0.91 and a concurrence of *C*=0.90. These are among the highest values ever reported for QD-based entangled photon sources. Most remarkably, the whole set of measurements draws an unambiguous conclusion that we have obtained a large ensemble of entangled photon emitters on a single semiconductor wafer, with almost 100% of QDs in the sample having fidelities *F*>0.5. Moreover, a great fraction of QDs are expected to exhibit high fidelities *F*>0.8 without any post-growth tuning.

We envision that a number of key enabling quantum photonic elements can be practically implemented by using this novel material system. A particularly important example is a quantum repeater as the backbone for long-range quantum communication. One requirement for a quantum repeater is the storage of entangled photon pairs within a quantum memory at a millisecond timescale^39^,^40^. Promising candidates for exceedingly long coherent storage are ensembles of laser-cooled atoms^41^,^42^,^43^,^44^, single trapped atoms^45^, impurity-doped crystals^46^,^47^ and optomechanical systems^48^. A potential material system for the storage of photons in the telecom wavelength range is erbium-doped Y_2_SiO_5_ (ref. 49). However, the storage capabilities of the latter do not yet exceed the nanosecond timescale. Thus, the QDs presented in this work are specifically tailored to match the optical transitions of rubidium that is among the most mature storage candidates. Thereby, we envision a hybrid quantum repeater that incorporates QD-generated entangled photon qubits that can be mapped reversibly in and out of a rubidium vapour-based quantum memory.

To fully reach that goal, however, high source brightness and photon indistinguishability also have to be ensured, for example by implementing QDs into microcavities. Additionally, entangled photons from different sources have to be spectrally matched to meet the requirements for quantum interference. Different strategies have been experimentally implemented to address this issue^50^,^51^. Furthermore, efficient frequency conversion of the photons after storage to the telecom wavelength range would be desirable for practical long-range quantum communication.

During the preparation of the manuscript we noted a similar work by Huber *et al*.^52^ that was executed at the same time. Both works are rather complementary to each other: while their focus lies on high entanglement fidelities and good indistinguishability values of a few selected dots, we show an exceptionally high yield to obtain strongly entangled photon sources for rubidium hybrid systems.

## Methods

### Optical excitation

The photoluminescence experiments were conducted at *T*=4 K by placing the sample in either a helium bath or a helium flow cryostat. As excitation laser for the above band and two-photon excitation, a pulsed Ti:Sa laser with 76 MHz repetition rate was used, which generated pulses with a duration of 3 ps. To spectrally narrow the laser pulse it was sent to a home-built pulse-shaping setup before it was coupled into a single-mode fibre. The excitation laser was then sent to the sample in the cryostat using a beam sampler and focused by a lens or an objective that was also used for the collection of the QD emission. We used half-ball solid immersion lenses to increase the photon collection from the sample. The fluorescence signal was coupled into a polarization-maintaining single-mode fibre. To suppress the resonant laser background, two consecutive tunable notch filters were employed before signal detection with a spectrometer.

### Correlation measurements

To measure entanglement, the collected light from the QD was split by a 50:50 beam splitter into two arms, each containing a quarter-wave plate, a half-wave plate and a polarizer. The two beams were then coupled into polarization-maintaining single-mode fibres. After eliminating the residual laser using notch filters, each light path was fed into a monochromator to select the XX or X transition, respectively. The streams of photons were then detected by avalanche photodiodes, whose signals were processed by a time-correlated single photon counter. The integration times in the presented measurements ranged from 20 to 30 min for each correlation measurement, with count rates in the range of 2,500 c.p.s. up to 5,000 c.p.s. and a time binning of 64 ps.

### Characterization of optical properties

We measured the FSS of the sample by rotating the half-wave plate in the entanglement measurement setup by *α* while rotating the quarter-wave plate by 2*α*. By obtaining high-resolution spectra for multiple values of *α* it was possible to fit the emission lines and determine an oscillation amplitude of the peaks spectral centre position with sub-μeV accuracy that corresponds to the FSS. The FSS in the InAs/GaAs QD reference sample was determined using the same method, but simply by rotating a half-wave plate in front of a linear polarizer. The lifetimes *T*_1_ were obtained using an avalanche photodiode with a short response function that was measured by using 3 ps laser pulses to be FWHM ≈100 ps. The measured response function was used to obtain the convoluted theoretical fits.

### Data availability

The data that support the findings of this study are available from the corresponding author on reasonable request.

## Additional information

**How to cite this article:** Keil, R. *et al*. Solid-state ensemble of highly entangled photon sources at rubidium atomic transitions. *Nat. Commun.* **8,** 15501 doi: 10.1038/ncomms15501 (2017).

**Publisher's note:** Springer Nature remains neutral with regard to jurisdictional claims in published maps and institutional affiliations.
